# CATO: Wake-Up reCeiver-bAsed communicaTiOn for Batteryless Devices

**DOI:** 10.3390/s25226813

**Published:** 2025-11-07

**Authors:** Sayedsepehr Mosavat, Johannes Göpfert, Bernd-Christian Renner, Pedro José Marrón, Matteo Zella

**Affiliations:** 1 Department of Engineering and Computer Science, Niederrhein University of Applied Sciences, 47805 Krefeld, Germany; matteo.zella@hsnr.de; 2 Networked Embedded Systems Group, Faculty of Computer Science, University of Duisburg-Essen, 45127 Essen, Germany; pjmarron@uni-due.de; 3 Institute for Autonomous Cyber-Physical Systems, Hamburg University of Technology, 21079 Hamburg, Germany; johannes.goepfert@tuhh.de (J.G.); christian.renner@tuhh.de (B.-C.R.)

**Keywords:** ultra-low-power networking, MAC protocol, neighbor discovery protocol, batteryless system design, wake-up receiver, energy harvesting

## Abstract

Batteryless devices offer an unparalleled opportunity for long-term, low-maintenance, and sustainable operation. These opportunities are especially attractive in the context of the Internet of Things (IoT). Such devices, however, rely on a stringent energy budget for their operation. As a result, batteryless devices often operate intermittently. Therefore, energy-intensive functionalities such as wireless communication, though valuable in practical applications, are still a significant challenge to realize on such devices. This work proposes wake-up receiver-based solutions for facilitating energy-efficient communication among batteryless, energy-harvesting devices. As a foundation for realizing wireless communication, we introduce BEWARE-MAC, a MAC protocol that exploits the capabilities of the underlying WuR to enable efficient message exchanges among batteryless devices. To demonstrate the broadcast capabilities of BEWARE-MAC, we propose WEND, a WuR-based neighbor discovery protocol that can operate on intermittently powered, batteryless devices. Finally, we present an evaluation of the proposed protocols using both experimental and simulation-based results. Our results suggest that BEWARE-MAC can improve the goodput of energy-constrained devices by up to 61.1%.

## 1. Introduction

Recent advances in low-power electronics and energy harvesting technologies have enabled the introduction of batteryless devices that store ambient energy in a small energy buffer and use the accumulated energy to carry out various tasks. Such tasks cover multiple applications, such as sensing [1,2,3], entertainment [4], communication [5], streaming [6], and inference [7]. These applications were, in turn, made possible by the advancements made in the areas of software [8,9,10,11,12,13,14,15,16], hardware [17,18,19,20], and supporting tools [21,22,23,24,25,26] for batteryless devices. Nonetheless, the fact that batteryless devices often operate intermittently due to their small energy storage elements and the dynamic nature of harvestable energy still makes it challenging to utilize such devices like their battery-operated counterparts, which often have less stringent energy budgets but are hard to maintain on large scales over long periods.

In order to employ batteryless devices in real-world applications, a crucial yet challenging aspect that must be addressed is the communication capabilities of such devices. One possibility to address this facet is to employ backscatter communication [27]. Although backscatter communication could provide a low-power means of communication to batteryless devices, it relies on some form of existing infrastructure. Not only does such an infrastructure require appropriate deployment and maintenance, but it also imposes limitations, such as the scalability of the network of batteryless devices [28,29].

**Figure 1:**
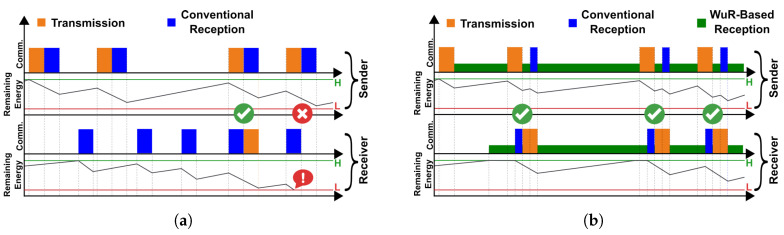
(**a**) Conventional communication using active radios requires synchronization between the sender and receiver. To achieve this goal, a receiver must consume considerable energy for idle listening. This behavior can introduce a prohibitively high energy overhead into a batteryless device, resulting in inefficient communication among such devices. (**b**) Wake-up Receivers (WuRs) enable efficient, low-power monitoring of the wireless channel. Thus, employing WuRs in the design of batteryless devices offers numerous advantages, such as low energy overhead, low communication latency, and high scalability.

An approach to enable self-sustained communication among batteryless devices is to employ RF transceivers to carry out active communication. However, as depicted in Figure 1a, wireless communication using active radios is not only an energy-hungry operation, it also introduces issues such as synchronization among the devices. Therefore, this facet still requires further investigation in the context of batteryless, energy-harvesting sensor nodes [30]. Novel approaches using active communication that enable the efficient use of the limited energy budget of such devices would facilitate their use in large-scale and sustainable deployments in a low-maintenance manner.

**Contributions**: In this work, we aim to address the challenge of energy-efficient wireless communication among batteryless devices by employing Wake-up Receivers (WuRs) to aid the communication among the nodes. As depicted in Figure 1b, the use of wake-up receivers and active communication offers the possibility of monitoring the wireless channel efficiently. Ultra-low-power monitoring of the wireless channel by devices, combined with active communication, removes infrastructure-related constraints on the system, such as reliance on ambient RF sources in backscatter communication, and also enables the use of diverse network topologies. It also minimizes the assumptions that might otherwise be required for the network’s efficient operation, such as persistent timekeeping. In this work, we propose a novel approach to exploit the capabilities of address-based WuRs to design MAC-layer mechanisms that aim to increase communication efficiency among energy-constrained devices with varying available energy levels. In summary, our proposed approach entails the following key contributions:

1. We propose BEWARE-MAC (BattEryless WAke-up Receiver-Enabled MAC), a Wake-up receiver-based MAC protocol. BEWARE-MAC is specifically designed to utilize the addressing capabilities of WuRs to improve communication performance among energy-harvesting, batteryless devices. This protocol has mechanisms to increase the probability of successful one-to-one data transmissions among nodes that may or may not have the same available energy at their disposal while increasing fairness in both cases. Furthermore, it supports the communication of devices in various network topologies, such as star-topology and multi-hop networks. BEWARE-MAC can also transmit multicast and broadcast messages from one device to the rest of the network’s active nodes.
2. To validate the benefits of BEWARE-MAC for unicast communication, we implement it on hardware prototypes and provide an experimental evaluation of the workings of this protocol in practice. Moreover, we present a simulation-based evaluation of BEWARE-MAC to provide additional insights into its performance under various operation conditions.
3. To demonstrate the capabilities of BEWARE-MAC for broadcast communication in practice, we propose and experimentally validate WEND (Wake-up receiver-assisted Energy efficient Neighbor Discovery), a wake-up receiver-based neighbor discovery protocol. WEND facilitates the energy-efficient discovery of batteryless devices in close proximity to each other [31,32]. Therefore, it provides a foundation for real-world applications that require neighbor awareness in networks of batteryless devices.

## 2. Related Work

In this section, we review the existing literature relevant to the various aspects of the present work. Due to the large body of literature on topics such as MAC and neighbor discovery protocols in wireless sensor networks, we limit our focus to works relevant to batteryless systems.

### 2.1. Networking of Batteryless Devices

Bonito [30] is a connection protocol utilizing a model to estimate the charging duration of batteryless devices. The nodes then use this estimate to maintain a bi-directional connection over time. Although an influential work in the area of communication among batteryless devices, Bonito focuses on a two-device setting rather than networks consisting of several batteryless nodes. TRAP [33] exploits a side channel to share the energy status of batteryless devices before commencing backscatter communication. The prior exchange of energy status aims to increase the likelihood of successful communication. However, in contrast to our proposed solution, TRAP assumes the presence of facilities such as an RF carrier source and persistent timekeeping.

### 2.2. WuR-Based Works

Several other works deal with the various aspects of WuR-enabled devices for energy-harvesting devices in the existing literature. Works such as [34,35,36] provide mathematical models and theoretical treatments of aspects such as power analysis of WuR-based networks of energy-harvesting nodes, as well as solutions for multi-hop and mesh networking of such devices. Moreover, MAC protocols for data gathering in star-topology networks [37,38], as well as multi-hop packet forwarding [39,40], have been proposed by the community. In this work, however, we use energy storage elements with significantly lower capacities since large storage elements either require more harvestable energy from the environment or take longer to charge. Moreover, our MAC protocol exploits a novel WuR that allows the wake-up signals to piggyback short pieces of data without requiring the main transceiver to turn on. Greentooth [41] employs a dual-radio architecture to develop WuR-based MAC and physical-layer protocols to achieve communication among intermittently operating, batteryless devices. However, since it only considers a single-hop network topology, the proposed solution cannot be directly used for larger networks that mandate multi-hop communication among the devices.

### 2.3. Neighbor Discovery Protocols

Find [42] is a neighbor discovery protocol for batteryless, intermittent devices that employs random delays after device activation to increase the efficiency of neighbor discovery. Flync speeds up the neighbor discovery of Find by exploiting the powerline-induced flicker to synchronize the different devices. However, Flync relies on mains-powered ambient lighting, which might not always be available, and without Flync, Find suffers from relatively long discovery delays. The approach introduced in [43] relies on low-power sensing of the RF channel to enable a batteryless device to discover another nearby one. The proposed approach, however, assumes the availability of a persistent timekeeper. Moreover, the performance of this solution requires further investigation in scenarios involving larger networks of batteryless devices. Dual-Range Bootstrapping (DRP) [44] is a mechanism that enables an energy-harvesting device to join a short-range, multi-hop network. This is achieved by employing a single-hop, long-range wireless link (LoRaWAN) to request the timing and synchronization information of the network from a central device. However, relying on a single central device to synchronize the entire network may not be scalable in the long run. It could also introduce a single point of failure in an otherwise self-sustaining network of batteryless devices.

In the present work, we rely on off-the-shelf components to design and implement a topology-agnostic, WuR-based MAC protocol. Therefore, unlike the state of the art, BEWARE-MAC is more versatile in terms of the supported networking architectures. As a result, this work can lay the foundation for the realization of large-scale networks of batteryless devices. However, the fact that related state-of-the-art works were designed for fundamentally different networking scenarios and based on different assumptions makes a direct quantitative comparison with the present work a non-trivial challenge. Table 1 provides a comparison between the key features of the present work and the state of the art.

## 3. CATO

In this section, we describe CATO (wake-up reCeiver-bAsed communicaTiOn for batteryless devices), which is a protocol suite that provides the building blocks for the efficient networking of energy harvesting, batteryless sensor nodes. To this end, CATO consists of a WuR-based MAC protocol (BEWARE-MAC) and a neighbor discovery protocol (WEND). BEWARE-MAC provides mechanisms for efficient and fair communication among WuR-based batteryless devices and WEND uses this MAC protocol to enable neighbor discovery among such devices.

### 3.1. BEWARE-MAC

Our proposed MAC protocol aims to facilitate energy-efficient wireless communication among batteryless, energy-harvesting devices with heterogeneous energy profiles. Such varying energy profiles can result from deploying nodes in locations with inherently different energy availability. For instance, in the case of a photovoltaic energy harvesting modality, a node deployed next to a window might experience a significantly higher level of harvestable energy than a similar device deployed under indoor lighting conditions. We utilize the addressing capabilities of a WuR to design MAC-layer mechanisms that achieve the aforementioned goal. Although BEWARE-MAC mainly focuses on a unicast communication modality, it also offers multicast and broadcast messaging among the nodes of a network of batteryless devices. Our proposed protocols are based on the FH101RF RFicient wake-up receiver [45,46] developed by the Fraunhofer Institute for Integrated Circuits (Fraunhofer-Institut für Integrierte Schaltungen IIS, Erlangen, Germany).

To carry out unicast transmissions among two nodes, the source node first sends a wake-up message to the destination node’s wake-up address. Moreover, the wake-up signal is used to piggyback 6 bytes of data, including a *frame control* data structure and the wake-up address of the source node. The frame control data structure consists of several pieces of information, such as the frame type, if the frame is being retransmitted, and the energy storage level of the source node. The destination node will receive this wake-up signal if it has enough energy to be awake and is in the range of the source node, processes the received data, and if it has enough energy available, turns on its main transceiver in preparation for the reception of the data payload of the frame. If the destination node receives the data payload of the MAC frame successfully, it notifies the source node by an acknowledgment. On the other hand, if the source does not receive an acknowledgment in time, it proceeds with a number of additional attempts. If these retransmissions are also unsuccessful, the MAC layer signals the data transmission failure to the upper layers. Due to this retransmission mechanism, a single MAC frame might require more than one transmission on the wireless medium to be successfully delivered.

It is also worth mentioning that to reduce the probability of frame collisions on the RF medium, we implement a clear channel assessment mechanism in the MAC protocol. Accordingly, each wake-up signal is preceded by a brief period of listening to the wireless channel to detect nearby, ongoing communication. If the RF medium is deemed to be free, the device sends the relevant data immediately; otherwise, it will reassess the channel after a short backoff period and attempt to transmit frames as soon as the channel is free again.

In the following, we introduce two novel mechanisms to increase the energy efficiency and fairness of BEWARE-MAC among batteryless devices by exploiting the addressing capabilities provided by a WuR. These mechanisms are particularly beneficial in scenarios where the communicating devices do not have the same amount of harvestable energy at their disposal.

**Figure 2:**
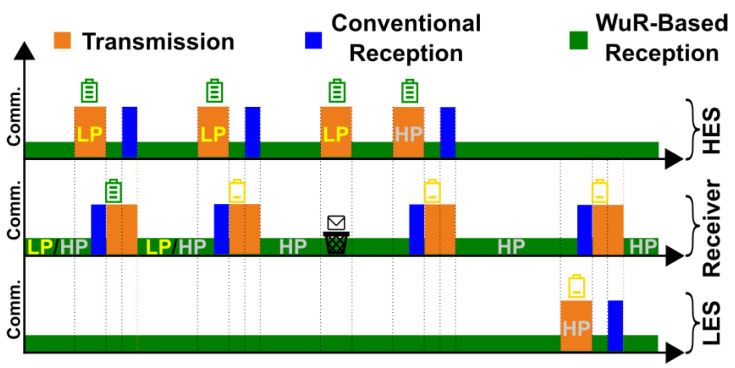
The prioritization mechanism allows a receiver to distinguish between low-priority and high-priority frames and only receive high-priority ones when its available energy is scarce. By exploiting data reception over the WuR, this decision can be made with minimal energy overhead and without requiring the main transceiver to turn on.

**Wake-up Priority:** The first mechanism we introduce in our MAC protocol is the *prioritization* of the wake-up channel. The primary objective of this mechanism is to prevent a sender with abundant available energy from monopolizing the limited energy resources of a receiver with constrained harvestable energy. The key idea of the prioritization mechanism is to utilize the addressing capabilities of the wake-up receiver to provide two types of frames for the unicast communication among devices. We refer to these frame types as low-priority and high-priority frames. The low-priority frames are used for communication attempts during periods of high energy availability, while the high-priority frames are used in cases where the remaining energy is scarce. The distinction between these two frame types enables a sender and receiver to attempt communication while considering their remaining energy. As illustrated in Figure 2, this prioritization allows a receiver to consider its remaining energy for listening to the incoming wake-up signals. Furthermore, a sender must also adhere to this prioritization scheme while transmitting data. The combination of these two requirements increases the likelihood of successful communication among batteryless devices that do not have the same amount of available harvestable energy.

**Figure 3:**
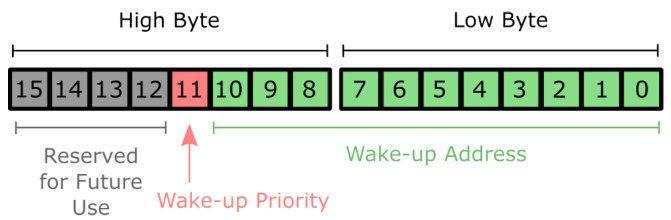
Wake-up addressing scheme.

To implement this mechanism in practice, we define two priorities for waking up a device and exploit the addressing capabilities of the FH101RF WuR to enforce the different wake-up priorities. As Figure 3 shows, the FH101RF receiver has a 16-bit address space for waking up specific receivers. We reserve the upper 4 bits for future use and utilize bit 11 to carry out the prioritization. Setting the priority bit implies a high wake-up priority, while clearing it implies a low one. The rest of the bits are used for enumerating the different sensor nodes of a network, ultimately resulting in an 11-bit address space for the specific devices.

At runtime, each node periodically checks the voltage of its energy storage element. If the remaining energy exceeds a pre-set threshold, the receiver cycles between the high and low reception priorities, responding to both. However, if the remaining energy is less than the threshold, the receiver will only respond to high-priority wake-up signals. It is worth mentioning that setting a particular reception priority entails updating the wake-up address of the WuR to reflect the corresponding priority bit (see Figure 3).

In the case of a sender, if its remaining energy at the time of a data transmission exceeds a pre-set threshold, it must first attempt to send the data using the low-priority address of the destination node. The sender can only use the high-priority address if the initial attempts with the low-priority address fail. However, if the sender does not have a high level of energy left at the beginning of the transmission, it can already use the high-priority address from the onset.

**Figure 4:**
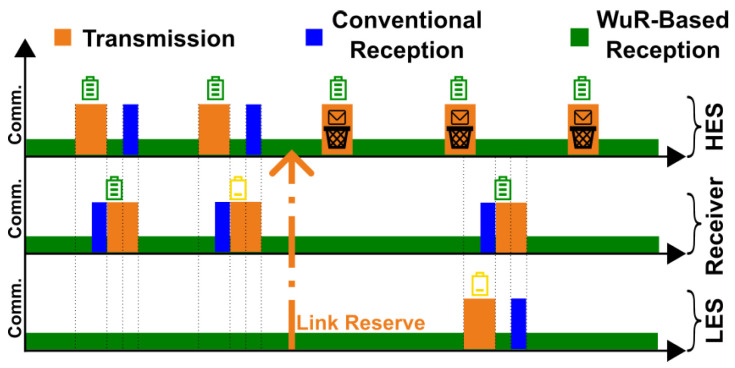
The link reservation mechanism relies on broadcast messages to notify nearby devices about the communication attempt of a low-energy sender (LES). As a result, a high-energy sender (HES) avoids addressing the designated receiver for a short period, resulting in the accumulation of energy on the receiver and, therefore, a higher chance of successful communication between the LES and the receiver.

**Link Reservation:** The second mechanism we introduce in our MAC protocol is *link reservation*, which aims to increase the likelihood of successful communication whenever a sender faces low available energy. The key idea of the link reservation mechanism is to exploit the broadcast capability of the wake-up receiver to notify devices in the vicinity of an energy-constrained node about ongoing communication events. While the wake-up prioritization mechanism, as discussed in the previous section, focuses on the cases in which *the receiver is experiencing low-energy conditions*, the link reservation mechanism focuses on *a sender that might not have much energy at its disposal for many transmission attempts*. As depicted in Figure 4, during a transmission attempt, if the sender experiencing a low energy availability does not receive an acknowledgment from the destination node after a few tries, it issues a link reserve to the rest of the network by broadcasting a frame containing the address of the destination node. All the other active devices in the vicinity of the sender will receive this message and avoid sending subsequent data to the same destination node for a specific duration. Consequently, the destination device will not receive wake-up signals from the rest of the network for a short period, increasing the likelihood of a successful transmission by the original sender. To broadcast a frame, a sender must use a hardware-defined wake-up sequence, specified by the FH101RF wake-up receiver, as the destination wake-up address. Upon reception of this pre-defined wake-up address, all devices that accept broadcast frames will be activated. As a result, the prioritization mechanism does not affect the broadcast messages, i.e., the devices will receive a broadcast message regardless of their reception priority.

**Wake-up Signal Data Rate:** The primary advantage of a WuR over a traditional transceiver in communication among batteryless devices is the possibility of listening to the wireless medium for incoming messages with almost negligible energy consumption. Therefore, the batteryless device can very efficiently wait for incoming messages, and only then is the transceiver required to be turned on to either receive lengthy portions of data at high data rates or transmit data to other devices. Yet, several performance trade-offs are inadvertently introduced due to the various techniques that WuRs use to reduce their energy consumption while listening to the wireless medium. One of the most notable trade-offs is the lower data rates supported by the WuR compared to traditional radio transceivers. As a result of the lower data rates supported by the WuRs, the sender of the wake-up sequence must stay in data transmission mode for a more extended period, resulting in higher energy consumption compared to data exchange with a higher data rate [34,35,36]. This phenomenon that we call *energy-debt* is crucial to consider when designing WuR-based networks of batteryless devices in which each node will not only be listening to the channel with a WuR but also sends wake-up messages to other nodes.

The FH101RF WuR receives the wake-up messages in two steps while listening on the wireless medium. The first step is the reception of a wake-up preamble, upon which the WuR listens for an additional, optional payload, including a wake-up address and user data. The FH101RF receiver supports various data rates for the preamble and payload data. The reason is that the preamble data rate dictates the energy consumption of listening for incoming wake-up signals, and a higher data rate will result in a higher energy consumption by the receiver while listening to the medium. On the other hand, since the payload data is longer than the preamble, increasing the payload’s reception data rate would make it possible to occupy the RF channel for a shorter time, leading to lower latency, energy consumption, and probability of interference.

**Figure 5:**
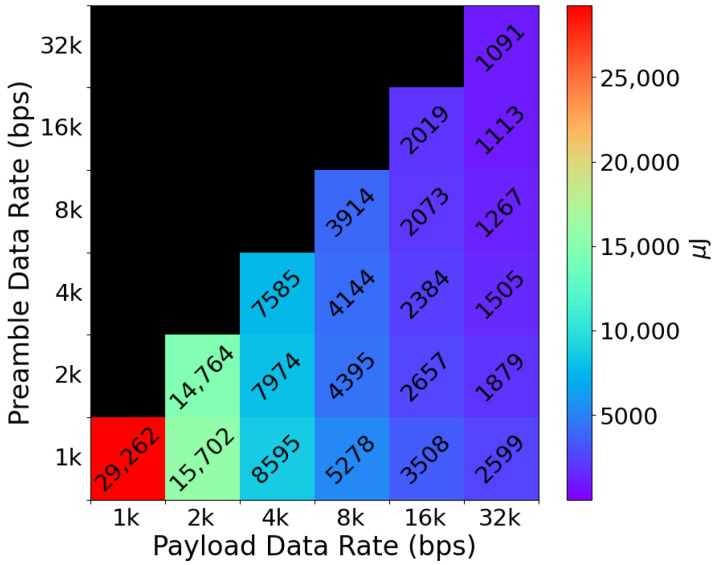
The energy consumption of transmitting a wake-up signal at different data rates. The payload data rate plays a more dominant role in the transmission energy than the preamble data rate since the payload is significantly longer than the preamble sequence.

Considering the discussions above, we employ a Keithley 2450 SMU (Keithley Instruments, Inc., Cleveland, OH, USA) for conducting experiments to measure the energy required to send wake-up signals by a sensor node (the hardware implementation of the sensor node will be discussed in detail in Section 4). In this case, the wake-up signal consists of the preamble followed by a 16-bit wake-up address. Figure 5 depicts the results of these experiments. As we can see in this figure, the wake-up signal’s transmission energy depends heavily on the payload data rate since this is the dominant part of the entire wake-up signal in terms of data length. Based on these results, we opt for a preamble and payload data rate of 1 kbps and 16 kbps for our MAC protocol, respectively, since these configurations provide a suitable trade-off between receiver and transmitter energy consumption as well as bit-error rate. It is worth mentioning that the black portion of the colormap in Figure 5 corresponds to data rate configurations that are not relevant in practice since setting the payload data rate lower than the preamble would result in inefficient operation in terms of latency and energy consumption.

### 3.2. WEND

BEWARE-MAC supports unicast, multicast, and broadcast communication. To demonstrate its broadcast capabilities, we utilize it to design WEND, a neighbor discovery protocol for batteryless devices that allows each device to discover the presence of other nodes in its vicinity. This section provides an overview of our proposed neighbor discovery protocol and its various features.

As soon as the start-up and initialization procedures of the peripherals of a sensor node are complete, the communication stack, including the MAC and neighbor discovery protocols, is initiated. The neighbor discovery protocol does not have any known neighbors at the beginning, leading each device to choose a short, random period to wait and then broadcast its presence to the rest of the network. Upon reception of a broadcast message from a neighbor, the receiving node adds it to the list of its local, known devices, noting the local power cycle and the timestamp of the incoming message. If the receiving node already has the device in its neighbor list, it only updates the power cycle and timestamp of the reception event. In this context, the local power cycle refers to a counter stored in the non-volatile memory of the MCU. This counter is incremented each time the batteryless device runs out of energy and has to carry out a startup routine after it has accumulated enough energy again. Since each batteryless device might experience power loss in a short period, it is essential that the devices maintain their knowledge of their neighbors after going through a power cycle. We, therefore, exploit the non-volatile FRAM of the microcontroller to store the neighbor list of each device. This is an important consideration to avoid unnecessary neighbor discovery messages each time a device runs out of energy since sending and receiving such messages too frequently would incur excessive energy overhead.

Furthermore, to increase the likelihood of finding at least one nearby device, a sensor node with no known neighbors broadcasts a *discovery request* message to the network to trigger other nodes in its vicinity to broadcast their presence. Upon reception of such a message, the receiving node waits for a random duration and then proceeds to broadcast its presence to the network. In this case, the random back-off period aims to reduce the probability of concurrent broadcast messages that would collide with each other.

To make neighbor discovery faster among physically adjacent devices of the network, each device includes a number of its known neighbors whenever it broadcasts its own presence. However, to minimize the propagation and persistence of stale neighbor information, these extra devices must be chosen from the ones the local node has discovered itself. In other words, suppose node A has received a discovery message from node B. Moreover, suppose that the discovery message B has sent also included information about the presence of a third device, which we call node C. Therefore, node A is now aware of the presence of node C, albeit indirectly, over the message received from node B. In this case, if A decides to broadcast its presence to the rest of the network, it will only include information about its own presence and node B, but not node C.

Finally, the neighbor discovery protocol periodically discards old entries that have not been updated for a specific duration in the same power cycle or have been received several power cycles ago. Moreover, the higher layers can provide hints to the neighbor discovery protocol about the staleness of a particular entry, e.g., after a transmission attempt to it has failed. The neighbor discovery protocol acts upon such hints after a specific entry has been reported to be outdated by discarding it from the list of known neighbors. These mechanisms will, therefore, increase the accuracy of the neighbor information throughout the operation of a batteryless device.

## 4. Hardware Implementation

**Figure 6:**
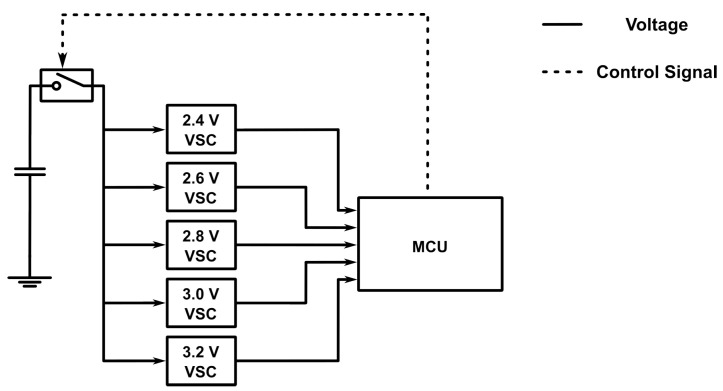
Simplified hardware design of the voltage supervisor subsystem. An analog switch is used to power an array of five voltage supervisor chips (VSCs) whenever a voltage reading is required. This subsystem enables the sensor node to measure the remaining energy stored in the energy storage element without incurring the overhead of using the analog to digital converter (ADC).

To experimentally validate the characteristics and performance of BEWARE-MAC and WEND, we implement them on a batteryless sensor node prototype. The hardware prototype used for the present work is a revision of a design proposed in a previous work [3]. Our prototype employs a low-power Texas Instruments MSP430FR5994 microcontroller (Texas Instruments Incorporated, Dallas, TX, USA) with an integrated FRAM non-volatile memory, which we use to persistently keep relevant pieces of information, such as neighbor lists, over the power cycles of the batteryless device. The microcontroller resides on an MSP-EXP430FR5994 LaunchPad Development Kit, while the rest of the sensor node is implemented on a custom expansion board. Each batteryless device harvests ambient energy using a Texas Instruments BQ25505 (Dallas, TX, USA) power management IC with maximum power point tracking. We set the lower and upper thresholds of the operation of the sensor node at about 2.15 V and 2.9 V, respectively. The sensor node stores the harvested energy in a combination of a 50 mF supercapacitor and a 100 uF MLCC. Each sensor node is also equipped with an FR101RF RFicient Basic (Fraunhofer-Institut für Integrierte Schaltungen IIS, Erlangen, Germany) [45,46] ultra-low-power wake-up receiver developed by the Fraunhofer Institute for Integrated Circuits (IIS). This innovative receiver facilitates selective wake-up of devices using a 16-bit address and can reach a sensitivity of −75 dBm on the 868 MHz frequency band that we use in this work. Moreover, we employ the Texas Instruments CC1101 (Dallas, TX, USA) sub-1 GHz wireless transceiver in each batteryless sensor node since it provides low-power wireless communication capabilities using a variety of modulations and data rates, which is, in turn, necessary for sending wake-up signals to the wake-up receiver. Each sensor node is equipped with an array of five ROHM Semiconductor BD52Exxx-series voltage detectors (ROHM Semiconductor, Kyoto, Japan) to monitor the remaining available energy efficiently. This voltage supervisor subsystem allows the sensor node to detect 2.4, 2.6, 2.8, 3.0, and 3.2 V voltage levels of the energy storage element without using the MCU’s analog-to-digital converter. To minimize the power consumption of the voltage supervisor, a Texas Instruments TS5A3166 analog switch (Dallas, TX, USA) is used to supply power to the voltage detectors only when a voltage reading is required and keep them unpowered otherwise. Figure 6 depicts a simplified overview of the hardware design of the voltage supervisor subsystem.

We utilize the microcontroller’s real-time clock (RTC) to implement a lightweight runtime that governs the operation of the entire software stack. To this end, the RTC is set to issue interrupts to the microcontroller at specific intervals. These intervals are determined by considering the readings of the voltage supervisor subsystem to assess the device’s available energy after each interrupt. A high energy availability results in the interrupt intervals being shorter, and if the remaining energy decreases, the RTC will also issue the next interrupt after a longer duration, allowing the sensor node to harvest more energy while in sleep mode. In this manner, each sensor node spends most of its operation in sleep mode and will be activated either upon an RTC interrupt or if the WuR receives a wake-up signal. In either event, the necessary actions, such as data processing or communication with other devices, are carried out, and the sensor node returns to sleep.

## 5. Evaluation

We conduct several experiments to characterize and evaluate the performance of the proposed approaches. To this end, we utilize the SOCRAETES solar cell emulator [25] to supply approximately 0.97 mW of harvestable power to each sensor node prototype at the maximum power point of the emulated IV curves. The supplied energy reproduces the available harvestable power under real-world conditions, helping to shed light on the performance of our proposed approaches under realistic conditions. In the following sections, we discuss the evaluated aspects.

### 5.1. MAC Protocol

We begin the evaluation by discussing the most relevant aspects of the MAC protocol’s performance. We compare the performance of different combinations of the MAC protocol’s two mechanisms against a baseline configuration that omits both. This baseline closely resembles state-of-the-art protocols such as W-MAC [47], and therefore serves as a reference point for evaluating the effectiveness of our proposed mechanisms relative to prior work.

#### 5.1.1. Experimental Results

As discussed in Section 3.1, each sensor node can change the priority of the wake-up signals it responds to by modifying its wake-up address and can also request other nodes to stop sending data to a particular destination for a period. To assess the impact of these mechanisms on data exchange in practice, we conduct an experiment consisting of three sensor node prototypes. One device acts as a data receiver, while the other two try to send packets to it whenever they have enough energy to do so. However, one of the senders has abundant available energy, while the other one experiences a shortage of harvestable energy. To simulate such a condition, we provide constant power to the device with high energy availability. The sender with the low energy availability, as well as the receiver, each harvests energy from a solar cell emulator. For the sake of simplicity, we refer to the sender with the high energy availability as the *High-Energy Sender (HES)* and the device with low energy availability as the *Low-Energy Sender (LES)* henceforward. Both senders attempt to transmit a packet to the receiver at short intervals, provided that they have enough energy for the transmission. The receiver also listens for incoming packets as long as it has enough energy to sustain its operation.

The experimental evaluation is based on a set of parameters that characterize the behavior of BEWARE-MAC. Selecting appropriate values for these parameters is a non-trivial task, as each introduces distinct trade-offs within the overall design space. The parameters used in the current evaluation were determined experimentally and are presented below.

The HES transmits frames every 2 s, and sustains this rate throughout the entire experiments. The LES, however, must adjust its transmission rate based on its remaining energy to avoid running out of energy unnecessarily. Therefore, it transmits frames every 2 s during high energy availability periods, while reducing to one frame every 5 and 7 s during medium and low energy availability periods, respectively. This behavior enables the LES to harvest more energy between subsequent frame transmissions if the energy conditions are not favorable. Each sender node can (re)transmit a frame for up to four times, with a random back-off period between 500 and 1750 ms between each transmission attempt. If the prioritization mechanism is activated, the HES can only use the high priority channel after the first two transmission attempts have failed. In contrast, if the LES is experiencing medium or low energy availability, it can alternate between the high and low-priority channels from the onset. If the link reservation mechanism is activated and a link reserve frame is received by a sender, it will pause transmitting frames to the designated destination for 15 s.

**Figure 7:**
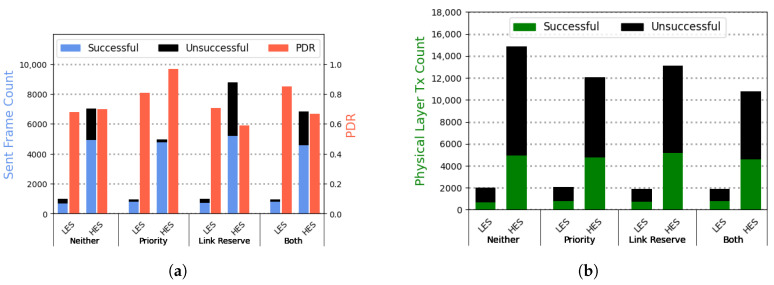
(**a**) Experimental results of the MAC protocol performance using different mechanisms. Using the wake-up prioritization and link reservation mechanisms, the LES can increase its probability of successful packet delivery while slightly decreasing the PDR of the HES. (**b**) Physical layer transmissions of the different MAC layer configurations.

We run experiments with the various possible combinations of the prioritization and link reserve mechanisms and collect data for 10 h for each setting. Figure 7a depicts the results of these experiments as seen by the higher layers of the communication stack, e.g., an application that uses the MAC layer for transmitting data. Moreover, Figure 7b shows the number of physical layer accesses by each sender to transmit a frame to the receiver. As these figures show, the prioritization mechanism tends to decrease the total number of transmitted MAC frames for the HES compared to the baseline (without either of the mechanisms active). This is because the HES must first try the low-priority wake-up channel, only switching to the high-priority one if the previous attempts were unsuccessful. Therefore, if the receiver is experiencing low energy availability, the HES must carry out more transmission attempts before it can use the high-priority channel to deliver a frame to the receiver. This will reduce the total number of frames sent, as the sender must wait for short periods before attempting to access the wireless medium again. However, the LES can successfully deliver more frames using the prioritization mechanism since it can use the high-priority channel for its transmissions whenever it experiences low available energy.

Regarding the link reservation mechanism, the results depicted in Figure 7 also show an improvement in the rate of successful frame transmission of the LES sender, compared to the baseline. This improvement is observed since issuing a link reserve request forces the HES to refrain from sending frames to the receiver temporarily, enabling the receiver to accumulate additional energy to accept more data before running out of power. This effect can be seen in the increased unsuccessful frames sent by the HES. In this case, the MAC layer immediately informs the higher layers about the reserved link and refuses to transmit it, resulting in an unsuccessful frame. Moreover, the refusal to send frames after a link reserve request is also evident from the reduced number of corresponding physical layer attempts compared to the baseline.

Finally, utilizing both mechanisms simultaneously could improve the likelihood of successful frame delivery for the LES while reducing the unsuccessful physical layer transmissions of the HES. This would, consequently, increase the energy efficiency of data transmission among batteryless devices, especially in scenarios where the devices do not have the same amount of available energy.

#### 5.1.2. Simulation-Based Results

Besides the hardware experiments, we tested our algorithm with a simulation framework based on NS-3 [48], modeling the sensor node’s energy consumption and the wireless communication channel. The mathematical model for wireless communication is based on the spectrum-aware channel and phy model presented in [49]. The model is based on the Shannon-Hartley theorem which describes the maximum data rate that can be transmitted over a communication channel with a given bandwidth in the presence of noise. This makes it possible to let the two modulation schemes, OOK and GFSK, used by the wake-up receiver and transceiver coexist and interfere with each other on the same physical channel.

As the energy consumption of the sensor nodes is crucial for the evaluation of the MAC protocol, we extended our simulation framework with a detailed energy model. The base model was developed in our previous work [50] and was extended with the energy consumption of the wake-up receiver and the transceiver.

Base of the model is a simple capacitor model which takes into account the current intake from the solar cell and power management IC as well as the energy consumption of the sensor node. The latter includes the energy consumption of the wake-up receiver and the transceiver as well as the energy consumption of the microcontroller and the sensor node’s peripherals. With this approach it is possible to assess parameter changes and more complex scenarios for the MAC protocol in a realistic way. Table 2 shows the current consumption values used for the simulation.

**Figure 8:**
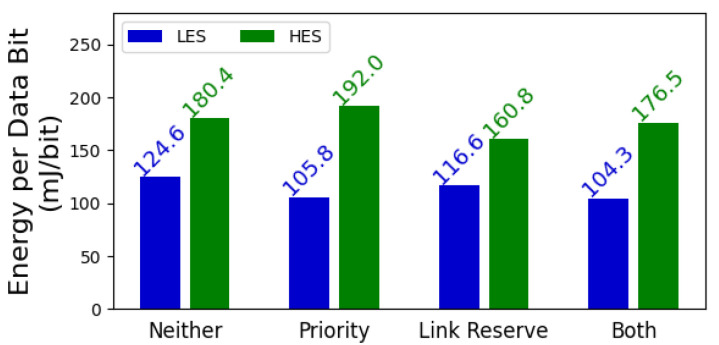
The energy required by BEWARE-MAC to transmit one bit of application data using various mechanisms. The prioritization and link reservation mechanisms generally decrease the energy demand for transmitting data, resulting in higher energy utilization.

To assess how the prioritization and link reservation mechanisms affect energy utilization, we use our simulations to calculate the required energy to transmit one bit of data using the MAC protocol. Figure 8 depicts the energy consumed per data bit using each mechanism. As apparent from this figure, both of the aforementioned mechanisms reduce the required energy per bit for the LES. This reduction is particularly desirable for the LES since it has a tighter energy budget than the HES. This will, in turn, result in higher energy utilization, particularly for devices that do not have an abundant amount of energy.

**Figure 9:**
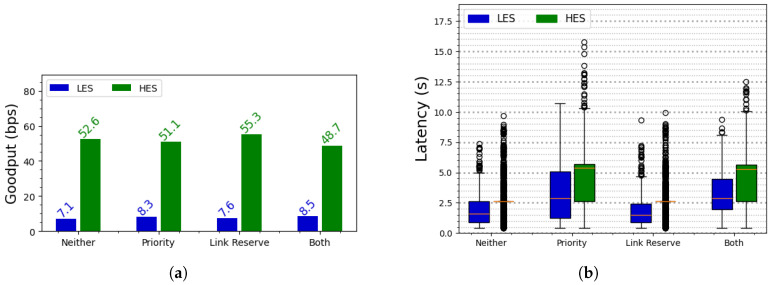
(**a**) Goodput achieved by the application under various MAC protocol mechanisms. For one LES and one HES, the prioritization and link reservation mechanisms generally result in higher goodput for the LES and lower goodput for the HES compared to the baseline case. (**b**) Latency under the various MAC protocol mechanisms. In general, enabling the prioritization mechanism leads to higher latencies because devices must attempt transmission on two different channels, thereby increasing the number of overall retransmission attempts required for successful frame delivery. The orange lines are a standard depiction for the median value.

Since goodput and latency are important metrics for the performance of any MAC protocol, it is crucial to evaluate the impacts of the prioritization and link reservation mechanisms on these metrics. Therefore, we measure these parameters while running an experiment over a period of 10 h with each mechanism. Figure 9 depicts the goodput and latency of BEWARE-MAC while running with each configuration. As depicted in Figure 9a, the application running on the LES achieved higher goodput when using the prioritization and link reservation mechanisms, whereas the HES generally exhibited lower throughput compared to the baseline case. This is an expected outcome since these mechanisms aim to increase the fairness of the communication when the senders have different amounts of available energy. On the other hand, Figure 9b illustrates the trade-off associated with using these mechanisms, namely an increase in communication latency. In the case of BEWARE-MAC, the latency results from the transmission time required for the WuS, the data, and random delays between iterations in case of failed attempts. Although the aforementioned mechanisms improve application goodput, they also lead to higher latency for both the LES and HES compared to the baseline case. The increase in latency is particularly significant when the prioritization mechanism is enabled. This elevated latency occurs because sender devices with high energy availability are required to utilize both the normal and high-priority channels when a transmission attempt begins. Furthermore, the HES generally exhibits higher latency than the LES, regardless of the employed mechanism. This is because an HES typically has sufficient energy to perform multiple retransmission attempts, whereas an LES may deplete its energy during an ongoing transmission. Consequently, the HES is more likely to experience a greater number of unsuccessful retransmissions per frame, resulting in a higher average latency. It is important to note that the values depicted in Figure 9 pertain to the goodput and latency of the MAC protocol *from the application perspective*. The application scenario described in Section 5.1 is designed such that the batteryless devices wait for a period after successfully sending a packet before transmitting a new one. Therefore, the goodput measured in this application scenario is not the maximum value attainable by the MAC protocol since the design of the application sets the upper bound of the goodput rather than the MAC protocol.

**Figure 10:**
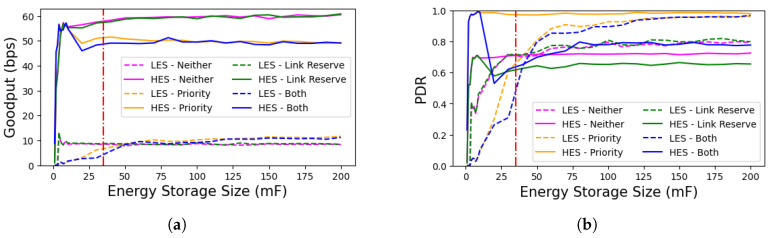
(**a**) Impact of the size of the energy storage on application goodput. In general, the goodput of the LES is best by using both prioritization and link reservation mechanisms, which is in direct contrast to the goodput of the HES. (**b**) The impact of the size of the energy storage element on the application packet delivery ratio. The HES has the highest PDR while using the prioritization mechanism since the receiver stays powered for a significant portion of the time using this mechanism. The HES can, therefore, eventually send its packet to the receiver successfully.

To evaluate the performance of BEWARE-MAC with different sizes of the energy storage element, we simulate the three-node scenario with various values. Figure 10 depicts the application goodput and packet delivery ratio (PDR) of the different MAC protocol configurations over a range of energy storage sizes. As apparent in Figure 10a, the LES generally achieves the highest goodput in cases that the prioritization mechanism is used (priority and both) and has the lowest goodput when not using either of the mechanisms. Similarly, as illustrated by Figure 10b, the LES generally achieves the best PDR in cases where the prioritization mechanism is used (priority and both). These results suggest that the proposed mechanisms effectively operate over a wide range of energy storage sizes as long as a minimum required energy storage value is available. This value, depicted with a vertical red line on Figure 10a,b, is the minimum energy storage size that would allow a device to perform all of the retransmission attempts without further energy input.

**Figure 11:**
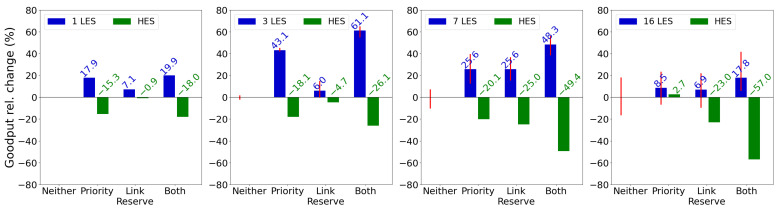
Relative change of the goodput for the different mechanisms of the MAC protocol with multiple LES devices. The red bars denote the range of goodput changes (min/max) among nodes, indicating whether mechanisms benefit all or only some LES devices. The link reservation mechanism peaks for seven LES devices, while the prioritization and link reservation mechanisms combined for three LES devices.

Finally, we evaluate the performance of the MAC protocol with more than two senders, increasing competition and evaluating the protocol’s ability to handle multiple energy-constrained senders. Figure 11 illustrates the relative change in mean goodput for different MAC protocol mechanisms across various numbers of LES devices (1, 3, 7, 16), compared to a baseline without any mechanism active. The red bars denote the range of goodput changes (min/max) among nodes, indicating whether mechanisms benefit all or only some LES devices. With one LES, as seen previously in Figure 9a, all mechanisms improve LES devices’ goodput, with both mechanisms together yielding a 19.9% increase but reducing the goodput of the HES by 18.0%. In this case, the link reservation mechanism, with an improvement of 7.1%, offers the least improvement to the LES. With three LES devices, enabling both mechanisms results in a 61.1% improvement for the LES devices and a 26.1% HES decline, the best improvement among 1–16 scenarios. For seven LES devices, the link reservation mechanism peaks at 25.6% w.r.t the improvement of the LES devices while reducing the goodput of the HES by 25.0%. When seventeen nodes (16 LES devices) try to communicate with one receiver, improvement is still 17.8% for the LES devices, with a 57.0% decrease in the goodput of the HES.

At this point, goodput improvement is not guaranteed for all LES devices, as indicated by the overlapping red bars. Overall, Figure 11 shows that BEWARE-MAC effectively accommodates multiple LES devices, with mechanisms enhancing goodput for nodes with lower energy budgets.

### 5.2. Neighbor Discovery Protocol

In this section, we present an experimental validation of WEND. We aim to demonstrate the broadcast capabilities of BEWARE-MAC and to evaluate the performance of WEND in practice and under realistic energy availability conditions. For this, we employ a set of five batteryless sensor nodes, each powered by a SOCRAETES solar cell emulator [25]. The emulators provide approximately 0.97 mW of harvestable power to each batteryless device at the maximum power point of the emulated IV curves. To simulate an intermittent operation scenario, each device periodically turns on an LED, which imposes approximately 6.74 mW of additional power consumption on the sensor node. This extra power consumption will consequently result in the depletion of the energy storage element. In this experimental evaluation, each neighbor discovery message includes up to one additional neighbor entry. Furthermore, each node removes outdated neighbor entries after five power cycles or, alternatively, after 120 s within the same power cycle, provided that no new updates have been received for the corresponding neighbor.

The first aspect we consider is the general behavior of the network of the batteryless devices while running the neighbor discovery protocol. In this case, we consider the operation of a network of devices with abundant harvestable energy so that the devices are, in effect, constantly powered without going through power cycles due to power loss. We carry out the experiment for 900 s to give the entire network enough time to stabilize. Each sensor node notifies a host machine during the experiment about the various protocol events using serial communication, and these events are later analyzed offline.

**Figure 12:**
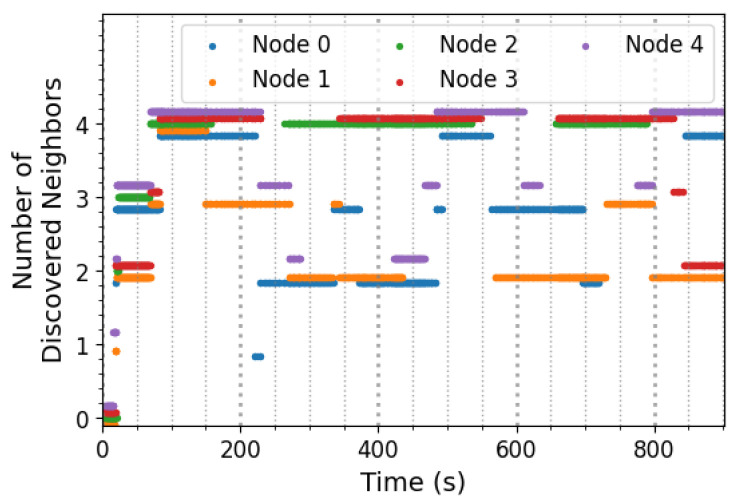
Sample behavior of the neighbor discovery protocol. After successful neighbor discovery, each device stores this information in non-volatile memory so that it can be used even after a power loss.

Figure 12 depicts the behavior of the 5-node network during a sample experiment. As it is apparent in this figure, each device successfully discovered a few of its neighbors soon after the beginning of its operation. The short discovery time stems from the fact that each device requests an advertisement round from the rest of the network briefly after it initially starts to operate if, at that point, there are no neighbors known to it. As described in Section 3.2 and shown in Figure 12, this mechanism enables the initial discovery of neighboring nodes within a short period.

Another feature of the behavior depicted in Figure 12 is that the devices do not lose their knowledge of their neighbors after a power cycle. This feature, as discussed in Section 3.2, contributes to the goal of energy efficiency since each node does not need to carry out a full sequence of neighbor discovery each time it experiences a power loss.

## 6. Discussion

In this section, we discuss aspects of the present work that can be relevant to the design of similar works that aim to realize efficient networks of batteryless devices.

**Advantages of active communication:** Relying on active communication avoids dependence on external infrastructure, enabling the development of systems with an emphasis on generality without imposing unnecessary constraints on the overall design. As a result, it will be possible to make batteryless networks easier to deploy in real-world scenarios. Employing WuRs in the design of batteryless, energy-harvesting devices makes active communication feasible in terms of power consumption. Consequently, such systems can be utilized in a wide variety of real-world applications with a minimum amount of tailor-fitting prior to deployment.

**Scalability:** The scalability of batteryless networks is essential for attaining solutions that can be employed in real-world scenarios. The topology-agnostic nature of BEWARE-MAC can aid with deploying various and flexible configurations of large networks of batteryless devices. Moreover, the two mechanisms we introduced in BEWARE-MAC make it possible to achieve this goal even under unfavorable or unpredictable energy conditions. Such self-organizing, self-sustaining batteryless networks that require minimum maintenance and intervention in the long run pave the path for achieving the vision of a ubiquitous, environmentally friendly Internet of Things.

**Advantages of WuR-based data transmission:** While WuRs enable monitoring the wireless channel efficiently and with low energy consumption, generating the wake-up signals is generally more energy-intensive for the senders due to the longer bit sequences required for WuRs. This applies to data parts explicitly intended for reception by the WuR, such as node IDs and additional data. However, this approach also offers vast possibilities for optimizing energy-efficient communication protocols so that such protocols can be effectively employed in ultra-low-power, batteryless networks. The mechanisms used in BEWARE-MAC showcase only a few such possibilities. In the future, we aim to explore further ideas for designing additional parts of the communication stack, with the ultimate goal of unleashing the full potential of WuRs for the design of sustainable and maintenance-free networks of batteryless devices.

**Performance in comparison to other SOTA works:** Carrying out fair and meaningful comparisons between works dealing with batteryless devices is a challenging task due to the hardware heterogeneity, different design assumptions, and the absence of a common evaluation methodology that can account for the large and complex design space of such systems. Although recent works such as [51] aim at providing a framework for the design and evaluation of batteryless systems, such frameworks still have not been widely adopted by the community. Therefore, a fair comparison of different works while accounting for their inherent dissimilarities, such as the energy harvesting modality, energy storage size, and energy consumption patterns still poses a challenge. Similarly, aspects like the networking topologies supported by different state-of-the-art works make a direct comparison challenging, since such aspects are a result of fundamentally different design decisions and operation assumptions. We therefore refrain from providing a quantitative comparison between the present work and SOTA works such as Bonito [30] and Greentooth [41], which are based on substantially different assumptions and hardware designs. Bonito provides a device-to-device communication solution between two batteryless, intermittently operating devices, and does not provide facilities for realizing larger networks of batteryless nodes. Likewise, the hardware design used for Greentooth only supports single-hop communication between batteryless devices and a central receiver, lacking the scalability for larger, potentially multi-hop networks. In contrast to these two works, BEWARE-MAC and WEND aim at providing a foundation for realizing scalable and flexible networks of batteryless nodes. This goal is achieved by enabling communication and neighbor discovery between batteryless devices without imposing limitations in aspects such as the network topology. These key differences between the present work and other SOTA works make a fair comparison among them prohibitively difficult, if not infeasible.

**Performance in multi-hop scenarios:** An important design goal of BEWARE-MAC is to support communication across diverse environments and network topologies. To achieve this, the protocol must enable both single-hop and multi-hop communication among batteryless devices. This is accomplished by integrating a traditional transceiver and a wake-up receiver into the design of each batteryless node. Consequently, every node can operate both as a data source and as a relay under the BEWARE-MAC protocol. The prioritization and link reservation mechanisms are particularly beneficial in the latter case, since a relay node must first function as a receiver and subsequently as a sender. Therefore, these two mechanisms can have a synergistic effect, improving the energy utilization of batteryless relay nodes. This is particularly true in dense network deployments, where devices may have differing amounts of harvestable energy available to them.

## 7. Conclusions

In this work, we have introduced BEWARE-MAC, a WuR-based MAC protocol for batteryless devices. BEWARE-MAC uses two mechanisms to increase the probability of successful unicast communication among the nodes even under unfavorable energy conditions. Furthermore, we introduced WEND, a WuR-based neighbor discovery protocol showcasing the broadcast-based capabilities of BEWARE-MAC. We provided an evaluation of our proposed solutions using experimental and also simulation-based results. Future work will focus on utilizing BEWARE-MAC and WEND to design real-world applications that rely on networks of batteryless devices.

## Figures and Tables

**Table 1:** Comparative overview of the key features of the present work and the state of the art.

Work	Network Topology Model	Transceiver	WuR	MCU Family
SNW-MAC [37]	Topology-dependent (Star-topology)	CC1120	Custom hardware	MSP430
Bonito [30]	Topology-dependent (Device-to-device)	Integrated 2.4 GHz Radio	Not used	nRF52
Greentooth [41]	Topology-dependent (Star-topology)	CC1101	Custom hardware + AS3932	MSP430
**Present work**	**Topology-agnostic**	**CC1101**	**FH101RF**	**MSP430**

**Table 2:** Current consumption values used for the simulation.

Component	State	Current Consumption
MCU	off	0 A
	sleep	30 μA
	active	1 mA
Wake-up Receiver	init	0.9 mA
	rx	3.5 μA
Transceiver	sleep	0.2 μA
	idle	1.0 mA
	rx	14 mA
	tx	13 mA

## Data Availability

The original contributions presented in this study are included in the supplementary material. Further inquiries can be directed to the corresponding author.

## Supplementary Materials

The following supporting information can be downloaded at: https://www.mdpi.com/article/10.3390/s25226813/s1, File S1: Experimental and simulation data obtained for the evaluation of the proposed protocols.

## Abbreviations

The following abbreviations are used in this manuscript:

WuR	Wake-up Receiver
LES	Low-Energy Sender
HES	High-Energy Sender
MAC	Media Access Control
MCU	Microcontroller Unit
PDR	Packet Delivery Ratio
OOK	On–Off-Keying
GFSK	Gaussian Frequency Shift Keying
LED	Light Emitting Diode
